# Density Functional Theory Description of Paramagnetic Hexagonal Close-Packed Iron

**DOI:** 10.3390/ma15041276

**Published:** 2022-02-09

**Authors:** Youngwon Choi, Zhihua Dong, Wei Li, Raquel Lizárraga, Se-Kyun Kwon, Levente Vitos

**Affiliations:** 1 Applied Materials Physics, Department of Materials Science and Engineering, Royal Institute of Technology, SE-10044 Stockholm, Sweden; zhihuad@kth.se (Z.D.); wei2@kth.se (W.L.); raqli@kth.se (R.L.); 2 State Key Laboratory of Mechanical Transmissions, College of Materials Science and Engineering, Chongqing University, Chongqing 400044, China; 3 National Engineering Research Center for Magnesium Alloys, Chongqing University, Chongqing 400044, China; 4 Department of Physics and Astronomy, Division of Materials Theory, Uppsala University, P.O. Box 516, SE-75121 Uppsala, Sweden; 5 Department of Physics, Pohang University of Science and Technology, Pohang 37673, Korea; sekk@postech.ac.kr; 6 Research Institute for Solid State Physics and Optics, Wigner Research Center for Physics, P.O. Box 49, H-1525 Budapest, Hungary

**Keywords:** hexagonal close-packed phase of iron, magnetic disorder

## Abstract

The hexagonal close-packed (hcp) phase of iron is unstable under ambient conditions. The limited amount of existing experimental data for this system has been obtained by extrapolating the parameters of hcp Fe–Mn alloys to pure Fe. On the theory side, most density functional theory (DFT) studies on hcp Fe have considered non-magnetic or ferromagnetic states, both having limited relevance in view of the current understanding of the system. Here, we investigate the equilibrium properties of paramagnetic hcp Fe using DFT modelling in combination with alloy theory. We show that the theoretical equilibrium c/a and the equation of state of hcp Fe become consistent with the experimental values when the magnetic disorder is properly accounted for. Longitudinal spin fluctuation effects further improve the theoretical description. The present study provides useful data on hcp Fe at ambient and hydrostatic pressure conditions, contributing largely to the development of accurate thermodynamic modelling of Fe-based alloys.

## 1. Introduction

Iron is one of the most common elements on Earth, constituting much of Earth’s outer and inner core. At atmospheric pressure, iron has two crystal structures; the body-centred cubic (bcc) and the face centred cubic (fcc) [1]. At high pressure, above ≈10 GPa, iron is observed in the hexagonal close-packed (hcp) structure [2]. The latter phase has been extensively studied due to its relevance to Earth’s inner-core research [3,4,5,6,7]. Nevertheless, the properties of the hcp phase of Fe at zero pressure are very important as well. Thermodynamic modelling requires information about hcp Fe to describe the phase diagram of Fe and Fe-based systems and corresponding physical quantities such as the stacking fault energy [8]. Since it is hard to synthesise hcp Fe at ambient conditions, one can estimate its properties by extrapolating the parameters of hcp Fe–Mn alloys to zero percent Mn content [9]. In cases such as this, where experiments can not be easily performed, quantum mechanics modelling based on Density Functional Theory (DFT) [10,11] becomes a useful approach to gain an insight into the properties of the system.

**Figure 1:**
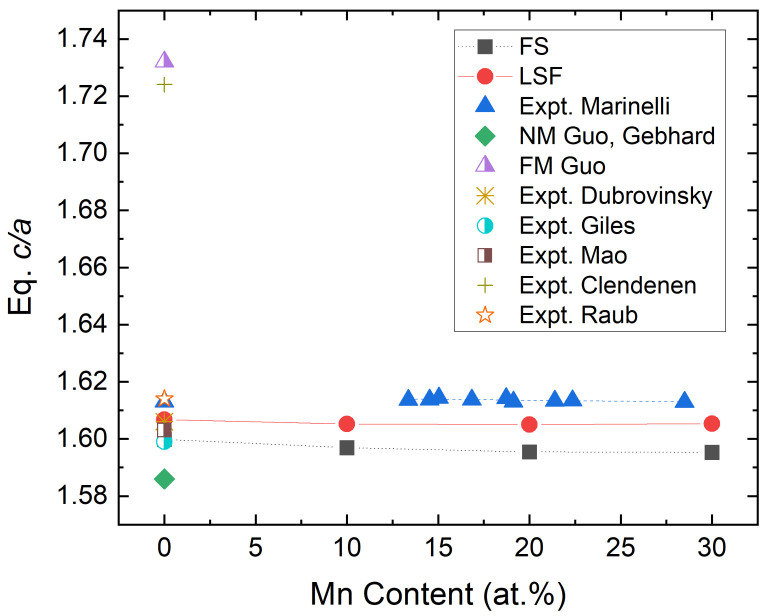
Equilibrium c/a versus Mn content of hcp Fe–Mn alloys in a paramagnetic state. Experimental values are taken from References [9,28,29,30,31,32]. The present theoretical data correspond to two calculation schemes: FS (floating spin calculations with usual disordered local magnetic moment model) and LSF (longitudinal spin fluctuations).

There are several studies on hcp Fe using DFT methodology, most of them focusing on the high-pressure regime such as the conditions of the Earth’s core [4,12,13,14,15,16,17,18,19,20,21,22,23,24,25]. Theoretical studies and predictions at ambient conditions often suffer from shortcomings. For instance, Guo et al. estimated the theoretical equilibrium hexagonal axial ratio c/a of hcp Fe depending on the adopted magnetic state [26]. By assuming a non-magnetic state for hcp Fe, c/a was predicted to be 1.586 [26,27], which is much lower than the value derived from the room-temperature data available for Fe–Mn alloys, 1.613 [9]. Instead, calculations for ferromagnetic hcp Fe predicted a larger value of c/a=1.732. Moreover, the pressure–volume relationship predicted from non-magnetic and antiferromagnetic calculations cannot properly reproduce the experimental data extrapolated at low pressure [22,23]. Figure 1 displays c/a versus Mn content for Fe–Mn alloys. The fact that non-magnetic and ferromagnetic c/a values differ from the extrapolated experimental values suggests that a proper account of the magnetic state is critical to improving the theoretical description of the properties of hcp Fe.

Iron is a magnetic metal, and its magnetic state has a decisive role in the crystal structure [6]. Both cubic phases of Fe at ambient pressure (α− and γ−Fe) are in fact stabilised by magnetism, the lack of which would result in the hcp structure observed in the other elements in group VIII (Ru, Os, and Hs). Despite theoretical predictions, Mössbauer effect measurements could not detect significant magnetic moments in hcp Fe [33,34]. Steinle-Neumann et al. addressed these inconsistent results by arguing that the hyperfine field from the core and valence contributions, which are used in experiments to detect magnetic moments, cancels each other so that no significant magnetic moment is observed [35]. In most of the early DFT studies under conditions of the Earth’s core, a non-magnetic state was assumed for hcp Fe. However, a more recent work demonstrated that there are non-vanishing local magnetic moments in hcp Fe even within the high-pressure and high-temperature regime [21]. The stacking fault energy in paramagnetic γ−Fe predicted finite magnetic moments at room-temperature near the fault plane, which is known to have a local hcp configuration [36]. Moreover, the Néel temperature of Fe–Mn alloys in the hcp phase was reported to be around or below room-temperature, depending on composition, and it decreases with decreasing Mn content [37]. Interestingly, the observation of unconventional superconductivity in hcp Fe in the pressure regime between 15 to 30 GPa [38] has long been associated with spin fluctuations [39,40]. All these findings suggest that a more reasonable model for the true magnetic state of hcp Fe at ambient conditions is likely to be the paramagnetic one with non-vanishing disordered local magnetic moments.

A recent study shows that going beyond DFT improves the description of hcp-Fe [41]. Namely, calculations based on dynamical mean-field theory (DMFT) predicted in good agreement with experiments the fundamental properties of hcp Fe, such as equilibrium volume, bulk modulus and equation of state. However, considering that, in general, DFT successfully describes the equation of state and equilibrium crystal structure of metals [42,43], the poor performance seen for hcp Fe and the apparent need to go beyond-DFT schemes raise several concerns.

Here, we present an investigation of the properties of paramagnetic hcp Fe at ambient conditions (room-temperature, zero and small hydrostatic pressure) by using DFT modelling in combination with alloy theory. We focus on the theoretical equation of state and c/a axial ratio of hcp Fe. We incorporate floating spin (FS) and longitudinal spin fluctuation (LSF) schemes in our calculations, which have been shown in the past to successfully predict the equation of state and planar fault energies in Fe–Mn [44] and Fe–Mn–Al (Si) alloys [45]. Finally, we show that our DFT approach, which takes into account the magnetic disorder, provides a highly accurate description of hcp Fe and can be used to derive reliable physical parameters for thermodynamic modelling of important Fe-based systems such as steels.

The present work is organised as follows: Section 2 describes the methodology used in this investigation, Section 3 contains results and discussions regarding structural and magnetic properties and equation of state, and finally, Section 4 presents the conclusions.

## 2. Methods

The Exact Muffin-Tin Orbitals (EMTO) method [46] is used to solve the Kohn–Sham equations in the DFT scheme [10,11]. Generally, DFT describes the lattice parameter of elemental metals with an error less than 2–3%, depending on the exchange–correlation approximation [42]. Here, we adopt the Perdew–Burke–Ernzerhof (PBE) approximation [47], which is one of the most accurate gradient-level exchange-correlation schemes for Fe. For comparison, we have also employed the quasi-non-local exchange correlation approximation (QNA) [48,49] that is known to predict highly accurate equations of state for bcc and fcc Fe [50]. The paramagnetic state is described by combining the disordered local magnetic (DLM) model [51,52] and the coherent potential approximation (CPA) [53,54], where Fe is split into two CPA components Fe${}_{1/2}^{↑}$ and Fe${}_{1/2}^{↓}$ with opposite magnetic moments. We consider static DLM (FS calculations) as well as thermal LSF effects. The latter effects have been included following the methodology described in References [36,50,55,56,57].

In the present investigation, we also discuss other contributions to the free energy such as phonons and entropy. Estimations of the lattice vibration effects are included using the Debye model [58].

We use the primitive unit cell to represent the hcp structure, consisting of $\vec{a}=a\left(1,0,0\right)$, $\vec{b}=a\left(-1/2,-\sqrt{3}/2,0\right)$ and $\vec{c}=c\left(0,0,1\right)$, where *a* and *c* are the parameters of the hcp structure, with two atoms at fractional coordinates, (0,0,0) and (0,2/3,1/2). The Brillouin zone (BZ) sampling is performed using 900 k-points in the irreducible part of the BZ.

## 3. Results and Discussions

### 3.1. Equilibrium Properties of hcp Fe

Table 1 shows structural properties; the equilibrium Wigner–Seitz (WS) radius and c/a using a static (0 K) DLM approach for paramagnetic hcp Fe. The static WS radius (2.547 Bohr) differs from the experimental one at 300 K by 0.072 Bohr. When correcting for the thermal expansion effect by using the theoretical thermal expansion coefficient [22], the error is reduced to 0.057 Bohr. This error corresponds to 2.2% and can be regarded as a general DFT error regarding the volume determination [59]. The static c/a is 1.590 and the deviation relative to the experimental value corresponds to 1.4%. By using the room-temperature experimental volume, the theoretical c/a increases from 1.590 to 1.600. We point out here that when the volume is fixed to the experimental value, the paramagnetic state (static DLM) possesses a finite local magnetic moment of ≈1 $\mu_{B}$. In other words, hcp Fe exhibits a paramagnetic state at ambient conditions with non-vanishing local magnetic moments, taking into account the LSF scheme further improves the prediction for the equilibrium volume and c/a. At 300 K, calculations accounting for LSF (but neglecting all other thermal effects) give 2.557 Bohr for the WS radius and 1.595 for c/a. If one adopts the experimental room-temperature WS radius (2.619 Bohr), with LSF at 300 K, one obtains 1.607 for the hexagonal axial ratio, so the error between theoretical and experimental values drops to 0.4%.

The magnetic moment obtained by including LSF is 1.49 $\mu_{B}$, which is similar to the value around a stacking fault in fcc Fe (local hcp environment) at room temperature (≈1.35 $\mu_{B}$) [36]. We notice that without considering LSF, the local magnetic moment around a stacking fault in fcc Fe is $0\;\mu_{B}$, and the local magnetic moment in bulk fcc Fe and hcp Fe are 1.2 $\mu_{B}$ and 0 $\mu_{B}$, respectively [60]. QNA results are also included in Table 1 for comparison.

We extend the above calculations to paramagnetic hcp Fe–Mn alloy. The results are shown in Figure 1, together with the available experimental data used to estimate the experimental c/a ratio for pure hcp Fe. We see that the FS values change slightly with the Mn content from 1.6 at 0 at.%Mn to 1.595 at 30 at.%Mn but the errors seen for the FS values relative to the experimental data do not change with Mn content significantly. Similarly to pure Fe, the LSF scheme improves the results for the Fe–Mn alloys as well and gives c/a ratios consistently closer to the experimental data compared to the FS results.

### 3.2. Magnetic Moments Dependence on c/a

**Figure 2:**
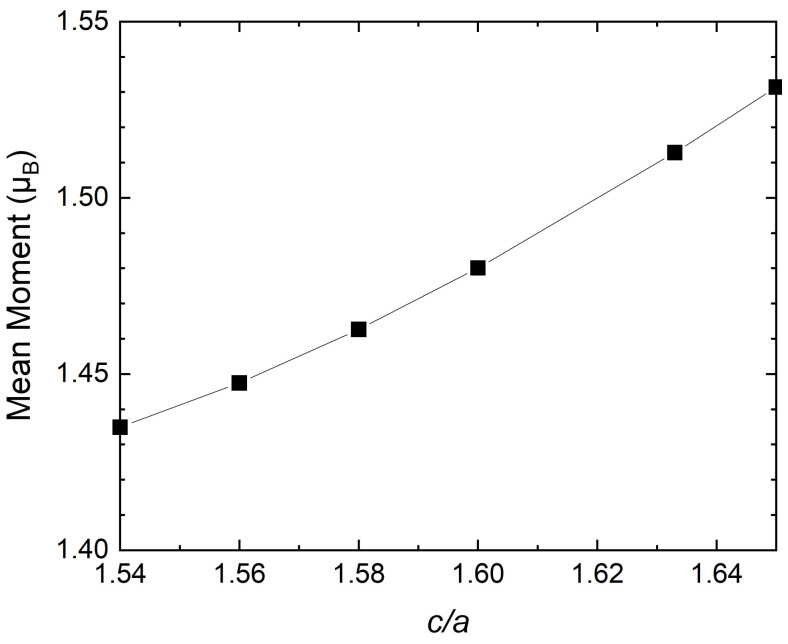
Mean magnetic moment versus c/a for hcp Fe obtained using the LSF calculations at 300 K and the volume fixed to the room-temperature experimental value (Wigner–Seitz radius of 2.619 Bohr [9]).

In order to understand the factors that lead to good agreement with experiments, we observe that the mean local magnetic moment calculated using the LSF scheme at 300 K depends on c/a. For the definition of the mean moment within the LSF approach, we refer the reader to Reference [57]. Figure 2 shows that as c/a increases from 1.54 to 1.65, the mean (LSF) magnetic moment increases from 1.43 $\mu_{B}$ to 1.53 $\mu_{B}$. This phenomenon can be analysed from various perspectives. The mean distance between atoms in hcp unit cells with *a* and *c* parameters is expressed as

$$\frac{1}{2}\left(a+\sqrt{\frac{1}{3}a^{2}+\frac{1}{4}c^{2}}\right).$$

By plotting this expression as a function of c/a under constant volume conditions one can see that c/a decreases in a nearly parabolic manner as it reaches 1.63 and increases again as c/a passes by 1.63. This means that the mean distance between atoms decreases as c/a increases from 1.54 towards the ideal 1.63 value. Therefore, taking into account that a larger mean distance usually corresponds to larger magnetic moments [45], the increase in the moment depending on c/a is unlikely to correlate with the atomic distance.

**Figure 3:**
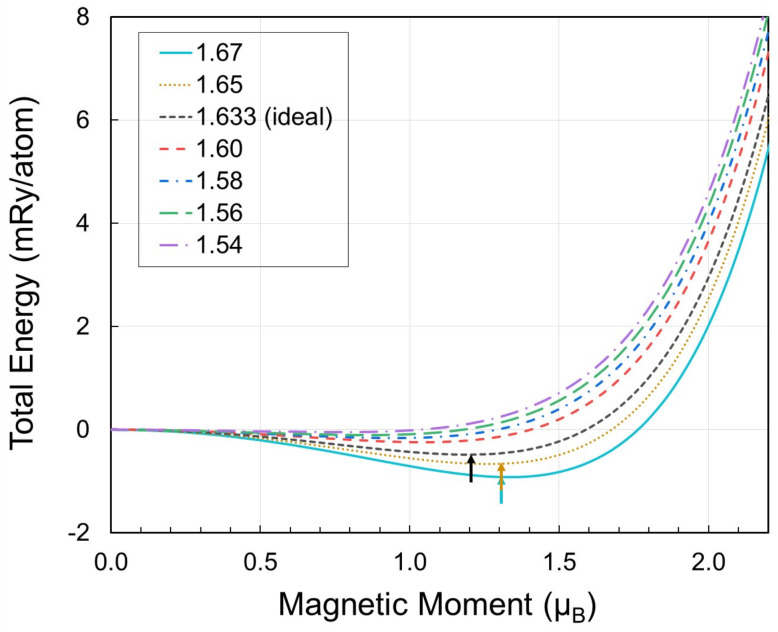
Total energy vs. local magnetic moments for paramagnetic hcp Fe for different c/a values (shown in legend) and volume fixed to the room-temperature experimental volume (WS radius of 2.619 Bohr [9]). The arrows indicate the minimum for each curve (cyan: 1.67, red brown: 1.65, and black: 1.633 (ideal)).

Next, we verify the above statement from an energy point of view by monitoring the energy-magnetic moment distribution for different c/a, as shown in Figure 3. As c/a decreases, the spontaneous paramagnetic moment (corresponding to the local minimum of the energy vs. moment) gradually disappears. Even though temperature can still induce finite disordered moments at low c/a, the mean value will be shifted towards smaller values due to the shifted local energy minimum. This is an interesting example that shows the coupling between local magnetic moments and c/a.

### 3.3. c/a Dependence on WS Radius

**Figure 4:**
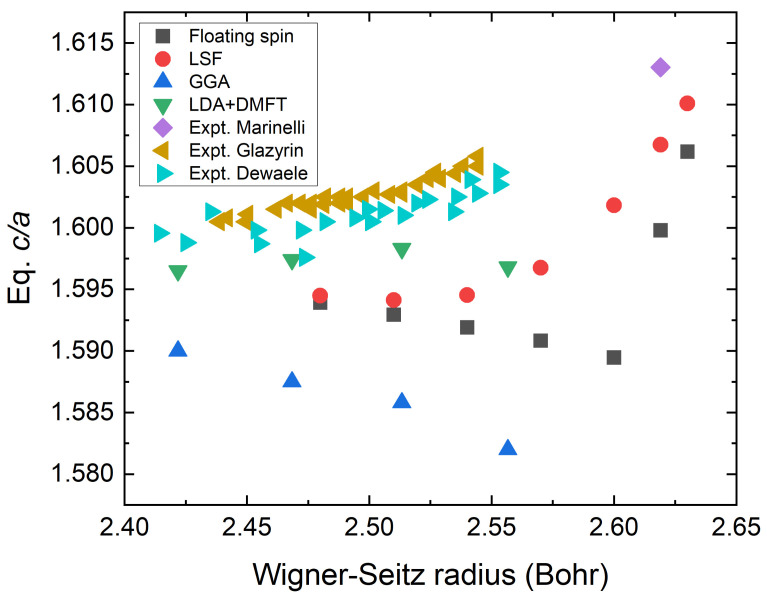
Equilibrium m c/a versus WS radius adopting different theoretical methods versus the experimental values. The diamond symbol indicates the extrapolated data from Fe–Mn alloys at zero Mn content [9]. Other experimental values (cyan and light brown triangles) are from Glazyrin et al. and Dewaele et al., Reference [61] and Reference [62], respectively. Floating spin means a usual disordered local moment model without applied LSF and LSF means the value where LSF is applied. The GGA and LDA+DMFT values are from Reference [41].

We also examine the equilibrium hexagonal c/a ratio in terms of the WS radius. In Figure 4, we compare our results with data from the literature. It is shown that the equilibrium c/a ratio in experiments slightly increases as the WS radius increases, as indicated by the right-pointing and left-pointing triangles. As the WS radius increases from 2.425 to approximately 2.55 Bohr, the equilibrium c/a value increases from 1.599 to 1.603 (0.032/Bohr) and finally reaches 1.613 at the experimental WS radius expected from Fe–Mn alloys by extrapolating the Mn content to zero (2.619 Bohr). This suggests that the extrapolation offers a good estimate for hcp Fe. Our theoretical value denoted by FS decreases with increasing WS radius, which is in good agreement with the previous calculations [41]. On the other hand, the values calculated from the method that incorporates the LSF scheme increases in a parabolic manner with increasing WS radius. This behaviour is clearly related to the non-vanishing magnetic moment in the LSF scheme. We notice that the value from the FS calculations also increases rapidly when a static DLM magnetic moment emerges at about 2.63 Bohr.

### 3.4. Effects of Magnetic Moments on c/a

**Figure 5:**
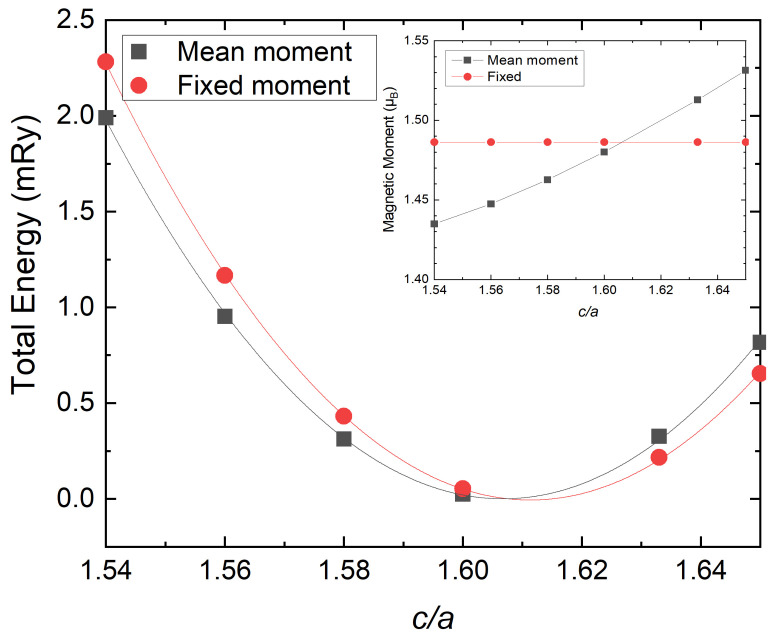
Total energy versus c/a for fixed moments (red circle) and variable ones (black square) calculated by the LSF scheme for volume fixed to the room-temperature experimental value (WS radius of 2.619 Bohr) [9]. The inset indicates the corresponding magnetic moment for each c/a.

In the following, we estimate the effect of the local magnetic moment on the equilibrium c/a obtained by the LSF scheme. This is performed in two steps. First, we fix the magnetic moment at a constant value (corresponding to the equilibrium c/a in the LSF scheme, see inset in Figure 5) for all c/a ratios and observe its effects on the equilibrium c/a. Then, we vary the size of the magnetic moment from 0 to 3.0 $\mu_{B}$ and see how the equilibrium c/a changes. These two steps can be regarded as a fairly complete picture for the magnetic moment effects on the equilibrium c/a. Figure 5 (main panel) shows that the minimum of the total energy curve moves towards larger c/a values when we fix the magnetic moment. The insert of Figure 5 shows that the fully self-consistent mean moment increases almost linearly with c/a. Hence, when fixing the moment to the reference point of the equilibrium c/a value, the actual magnetic moment is larger than the self-consistent moment for c/a less than the equilibrium value and smaller for larger c/a values. We would like to stress here that we focus on the total energy–magnetic moment relationship rather than the physical aspect of the free energy, which is minimised at the thermo-magnetic moment. As a result, when fixing the moment, the equilibrium c/a changes from 1.607 to 1.611.

**Figure 6:**
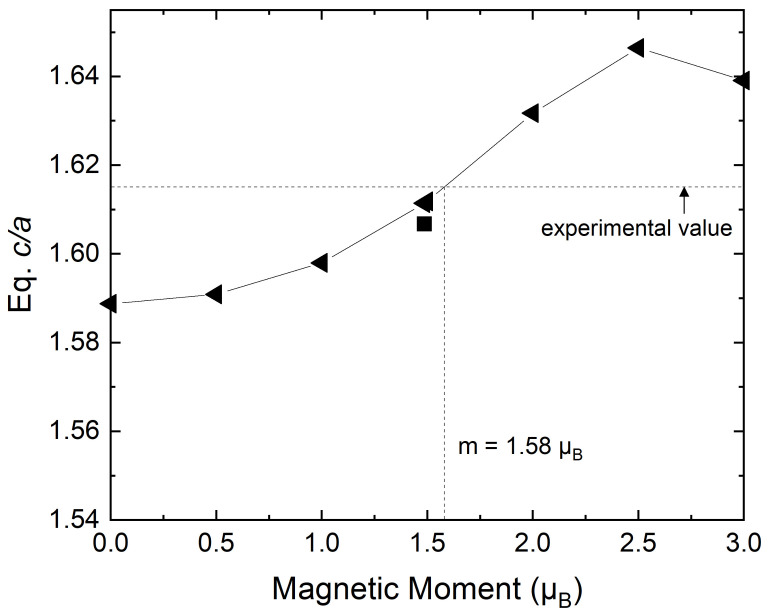
Equilibrium c/a versus magnetic moment of hcp Fe when we fix the local magnetic moments (left-pointing triangle). The square indicates the equilibrium c/a determined from the mean moment description shown in Figure 5 (black square).

Figure 6 illustrates how the equilibrium c/a changes as a function of the fixed local magnetic moment. As the moment increases from 0 to 2.5 $\mu_{B}$, the equilibrium c/a increases from 1.589 to 1.646, meaning about 0.023 per 1 $\mu_{B}$. We notice that at a magnetic moment of 1.58 $\mu_{B}$, we reach the experimental equilibrium c/a. Above 2.5 $\mu_{B}$, the equilibrium c/a decreases with the local moment and reaches 1.639 at 3.0 $\mu_{B}$.

### 3.5. Other Effects on c/a

Thus far, we have only considered the magnetic randomness and LSF effects. Next, we examine other thermal contributions to the equilibrium c/a. Firstly, we examine the phonon contribution to equilibrium c/a. Since a direct calculation of the phonons for a paramagnetic state can be cumbersome, here we resort to the Debye model to estimate the lattice vibration effects [58]. Following the approach in Reference [43], first we calculate the elastic constants for hcp Fe using the room-temperature experimental volume. The elastic constants are shown as a function of the WS radius in Figure S1 in the Supplementary Material. Results are collected in Table 2. From the theoretical elastic constants, we obtain the sound velocities and consequently the Debye temperature ($\Theta_{D}$).

From the Debye temperature, we can obtain the vibrational free energy [3,63,64] as a function of c/a. Thus, one can trace how the free energy is affected by the lattice vibrational effects. We notice that the elastic constants were calculated for simplicity in the non-magnetic state, which, however, is estimated to lead to a small (less than 1%) error in the Debye temperature relative to the one computed for the paramagnetic state (not shown). We found that c/a does not significantly affect the Debye temperature. Increasing c/a from 1.54 to 1.67 changes $\Theta_{D}$ by 13 K, which is small (2%) compared to its value at ideal c/a, 623 K. Therefore, the phonon contribution to the equilibrium c/a is expected to be small. When we calculate quantitatively using the high-temperature expansion of the vibrational free energy as a function of $\Theta_{D}$, the equilibrium c/a changes by 0.001 (from 1.607 to 1.606). Hence, the explicit phonon contribution is expected to have a small effect on the equilibrium c/a.

Subsequently, we consider the magnetic entropy associated to the spin disorder [64] as

(1) $$S_{\mathrm{mag}}=k_{B}\sum_{I,i}c_{I,i}\ln\left(1+m_{I,i}\right),$$

where $k_{B}$ is the Boltzmann constant, $c_{I,i}$, is the concentration of species *i* at site *I*, and $m_{I,i}$ is the corresponding local magnetic moment. Adding the magnetic entropy turns out to improve the estimation of the equilibrium c/a. At the room-temperature experimental volume (WS radius of 2.619 Bohr [9]), the floating spin (DLM) and LSF calculations predict 1.607 and 1.608, respectively. Both values are slightly larger than the results obtained without the magnetic entropy (1.600 and 1.607, respectively). Therefore, the magnetic entropy term has a moderate impact on the theoretical c/a especially within the LSF scheme.

Since spin fluctuations make a sizeable effect on the equilibrium c/a, we examined the approximations used in the present spin fluctuations scheme, such as fluctuating medium approximation (FMA), one-shot from static equilibrium approach (OSA) and mean moment approximation [36,44,45,50,55,56]. When we lifted FMA and OSA, the magnetic moment at ideal c/a changed by 0.02 $\mu_{B}$, which is not negligible but results in a small effect on the equilibrium c/a according to the relationship between the equilibrium c/a and local magnetic moment in Figure 6 (<$\;10^{-3}$). Furthermore, when we lift the mean moment description, that is, when we describe the random magnetic state rigorously with the probability distribution in the range of 0 to 3.0 $\mu_{B}$ instead of using the mean magnetic moment, the equilibrium c/a decreases only by 0.002. Therefore, the mean moment description seems to be accurate enough for the present theoretical description of c/a. The electronic entropy contribution was also considered. However, we found that at the range of temperatures considered here, this term can be regarded to be negligible [65].

### 3.6. Equation of States of Paramagnetic Fe

In the following, we describe the equation of state of hcp Fe and estimate the pressure–volume relation for c/a=1.60 within the LSF and non-magnetic schemes. By using a simple cubic equation of state [66], where the energy is represented as a function of the atomic volume *V*

(2) $$E\left(V\right)=a_{0}+a_{1}V+a_{2}V^{2}+a_{3}V^{3},$$

we obtain $a_{0}$ = −2543.202 Ry, $a_{1}$ = −0.095 Ry/Bohr${}^{3}$, $a_{2}$ = 0.001 Ry/Bohr${}^{6}$, and $a_{3}$ = −0.55 $\times 10^{-5}$ Ry/Bohr${}^{9}$. The pressure volume relation is accordingly derived as

(3) $$-\frac{dE}{dV}=p\left(V\right)=-a_{1}-2a_{2}V-3a_{3}V^{2}.$$

This is equivalent to Birch–Murnaghan type function with $E_{0}$ = −2545.605192 Ry, $V_{0}$ = 70.1 Bohr${}^{3}$, $B_{0}$ = 217.4 GPa, and $B_{0}^{{}^{\prime }}$ = 9.9. Here, $E_{0}$ is the energy at the equilibrium volume, $V_{0}$, $B_{0}$ is the bulk modulus, and B${}_{0}^{{}^{\prime }}$ is the pressure derivative of the bulk modulus. According to the literature data [23], at 19 GPa, the volume was measured to be 70 Bohr${}^{3}$ corresponding to WS of 2.557 Bohr. With respect to the equilibrium volume reported in the literature (75.4 Bohr${}^{3}$), V= 70 Bohr${}^{3}$ corresponds to 93% compression. We observe that the same volume ratio is reached at 27 and 24 GPa pressures for non-magnetic and antiferromagnetic calculations, respectively [23]. Based on our paramagnetic equation of state, we obtain that a pressure of 19 GPa leads to 93% volume compression, which is in good agreement with the experiment. Hence, the paramagnetic state gives an improved description for the equation of state represented by the pressure–volume relation. This is further evidence supporting that the magnetic state for hcp iron is paramagnetic.

## 4. Conclusions

In summary, we have investigated the equilibrium properties of hcp Fe in the paramagnetic state. We show that the prediction of c/a becomes more accurate when assuming a paramagnetic state for hcp Fe with magnetic moments corresponding to about 1 $\mu_{B}$ than the values predicted when using non-magnetic or antiferromagnetic states. Furthermore, the paramagnetic state provides a better prediction on the pressure–volume data at the low-pressure region compared to the non-magnetic calculations. The Néel temperature of hcp iron is expected to be low judging from the Néel temperature of Fe–Mn alloys with 17–29 at.%Mn (230 or 240 K) [67,68], and calculated data for hcp Fe under pressure (69 K) [69]. Hence, the magnetic state for hcp Fe at ambient conditions is likely to be paramagnetic. We also provide the equation of state for the paramagnetic state. The current research provides a consistent DFT-level description of hcp Fe, which is needed for thermodynamic modelling of Fe and Fe-based alloys.

## Figures and Tables

**Table 1:** Equilibrium Wigner–Seitz (WS) radius and c/a of paramagnetic hcp Fe. The magnetic moments in LSF calculations correspond to the mean magnetic moment (see the definition of mean magnetic moment in Reference [45]). The equilibrium WS radius for PBE calculations at 300 K corresponds to the lattice parameter obtained from the thermal expansion coefficient of hcp iron, $\left(2\times 10^{-5}\;K^{-1}\right)$ [22]. Equilibrium c/a is also calculated for the experimental WS radius, 2.619 Bohr. The experimental volume and lattice parameters are extrapolated data measured for Fe–Mn [9].

	Wigner–Seitz	c/a	a	c	Magnetic
	Radius (Bohr)		(Å)	(Å)	Moments $\left(\mu_{B}\right)$
PBE (0 K)	2.547	1.590	2.461	3.911	0
PBE (300 K)	2.562	-	-	-	-
PBE (Expt. Vol.)	2.619	1.600	2.525	4.039	1.16
LSF (300 K)	2.557	1.595	2.468	3.936	1.05
LSF (300 K, Expt. Vol.)	2.619	1.607	2.521	4.051	1.49
QNA (0K)	2.561	1.587	2.476	3.929	0
QNA (300 K)	2.577	-	-	-	-
Expt. (300 K)	2.619	1.613	2.517 ± 0.002	4.06 ± 0.01	-

**Table 2:** The Debye temperature depending on c/a for volume fixed to the room-temperature experimental value (WS radius of 2.619 Bohr [9]). $c_{s}$, $c_{66}$ and $c_{44}$ correspond to the coefficient for isochoric, orthorhombic, and monoclinic distortion, respectively. $c_{13}$ and $c_{33}$ are obtained from $c_{S}$ and bulk modulus (*B*). $c_{11}$ and $c_{12}$ are obtained from $c_{13}$, $c_{33}$, and $c_{66}$. The Debye temperature is obtained from the sound velocities calculated from the elastic constants [43].

c/a	$c_{s}$	$c_{66}$	$c_{44}$	*B*	$c_{13}$	$c_{33}$	$c_{11}$	$c_{12}$	$\Theta_{D}$
	(GPa)	(GPa)	(GPa)	(GPa)	(GPa)	(GPa)	(GPa)	(GPa)	(K)
1.54	879.7	146.8	138.1	190.4	92.6	385.9	386.1	92.4	610
1.56	924.7	151.6	134.2	193.1	90.3	398.6	396.0	92.8	614
1.58	975.7	156.6	129.9	193.1	84.6	409.9	403.8	90.7	617
1.60	1012.7	161.5	125.9	190.8	78.3	415.8	408.5	85.6	619
1.633	1069.4	168.9	119.1	192.4	73.6	430.1	420.8	82.9	621
1.65	1099.7	173.3	115.9	194.7	72.5	439.0	429.0	82.5	623
1.67	1118.1	177.3	112.2	193.1	68.9	441.6	432.6	78.0	622

## Data Availability

The data presented in this study are available on request from the corresponding author.

## Supplementary Materials

The following are available online at https://www.mdpi.com/article/10.3390/ma15041276/s1, Figure S1: (a) Bulk modulus of hcp Fe as a function of theWigner–Seitz radius. (b) cS of hcp Fe as a function of c/a and volume. (c) c66 of hcp iron as a function of c/a and volume. (d) c44 of hcp iron as a function of c/a and volume.
