# Supplementary material for: Density Functional Theory Description of Paramagnetic Hexagonal Close-Packed Iron
Source: Materials (Basel). 2022 Feb 9;15(4):1276. doi: 10.3390/ma15041276 (PMC8879897; doi:10.3390/ma15041276)
Supplement: Supplementary file 1 [file materials-15-01276-s001.zip › materials-1520252-supplementary.pdf]

materials

Article

Supplementary Material: Density Functional Theory Description of Paramagnetic Hexagonal Close-Packed Iron

Youngwon Choi [1,*], Zhihua Dong [1,2,3], Wei Li [1,4], Raquel Lizárraga [1], Se-Kyun Kwon [5] and Levente Vitos [1,4,6,*]

1 Applied Materials Physics, Department of Materials Science and Engineering, Royal Institute of Technology, SE-10044 Stockholm, Sweden
2 State Key Laboratory of Mechanical Transmissions, College of Materials Science and Engineering, Chongqing University, Chongqing 400044, China
3 National Engineering Research Center for Magnesium Alloys, Chongqing University, Chongqing 400044, China
4 Department of Physics and Astronomy, Division of Materials Theory, Uppsala University, P.O. Box 516, SE-75121 Uppsala, Sweden
5 Department of Physics, Pohang University of Science and Technology, Pohang 37673, Korea
6 Research Institute for Solid State Physics and Optics, Wigner Research Center for Physics, P.O. Box 49, H-1525 Budapest, Hungary
* Correspondence: ywchoi@kth.se,levente@kth.se

Abstract: The hexagonal close-packed (hcp) phase of iron is unstable at ambient conditions. The limited amount of existing experimental data for this system has been obtained by extrapolating the parameters of hcp Fe–Mn alloys to pure Fe. On the theory side, most of the density functional theory (DFT) studies on hcp Fe have considered non-magnetic or ferromagnetic states, both having limited relevance in view of the current understanding of the system. Here, we investigate the equilibrium properties of paramagnetic hcp Fe using DFT modeling in combination with alloy theory. We show that the theoretical equilibrium c/a and the equation of state of hcp Fe become consistent with the experimental values when the magnetic disorder is properly accounted for. Longitudinal spin fluctuation effects further improve the theoretical description. The present study provides useful data on hcp Fe at ambient and hydrostatic pressure conditions, contributing largely to the development of accurate thermodynamic modelling of Fe-based alloys.

Keywords: hexagonal close-packed phase of iron; magnetic disorder

check for updates

Citation: Choi, Y.; Dong, Z.; Li, W.; Lizárraga, R.; Kwon, S.-K.; Vitos, L. Supplementary Material: Density Functional Theory Description of Paramagnetic Hexagonal Close-Packed Iron. *Materials* **2022**, *15*, 1276. https://doi.org/10.3390/ma15041276

Academic Editor: Aleksandr Oreshonkov

Received: 06 December 2021
Accepted: 01 February 2022
Published: 08 February 2022

Publisher's Note: MDPI stays neutral with regard to jurisdictional claims in published maps and institutional affiliations.

Copyright: © 2022 by the authors. Licensee MDPI, Basel, Switzerland. This article is an open access article distributed under the terms and conditions of the Creative Commons Attribution (CC BY) license (https://creativecommons.org/licenses/by/4.0/).

0.1. Elastic constants of hcp Fe

Finally, we present our results for the volume dependence of the elastic constants used for estimating the Debye temperature. The aim is to provide the data for thermodynamic modeling and verify the outcomes in Table 2 to examine the effects of phonons on determining the equilibrium c/a. Figure 6a-6d shows the bulk modulus, c_S, for isochoric deformation, c_{66}, for orthorhombic distortion, and c_{44}, for monoclinic strain as a function of atomic volume expressed as the Wigner–Seitz radius. It is shown that the bulk modulus decreases with increasing volume. It is 390, 245, and 121 GPa at 2.48, 2.58, 2.68 Bohr, respectively, for ideal c/a. Furthermore, c/a variation is negligible compared to the volume change for the bulk modulus. For c_S, c_{66}, and c_{44}, the elastic constants depend on c/a more significantly than the bulk modulus. c_S changes from 1146 to 1479 GPa as c_{66} varies from 193 to 232 GPa, and c_{44} changes from 184 to 147 GPa as c/a varies from 1.54 to 1.67.

c/a dependency of c_S and c_{66} is different from c_{44}. However, for all elastic constants, the volume dependence (decreasing) is the same. For example, for ideal c/a, the bulk modulus changes from 300 to 98 GPa, c_S from 1408 to 806 GPa,c_{66} from 222 to 128 GPa, and c_{44} varies from 157 to 89 GPa as the WS radius changes from 2.54 to 2.70 Bohr.

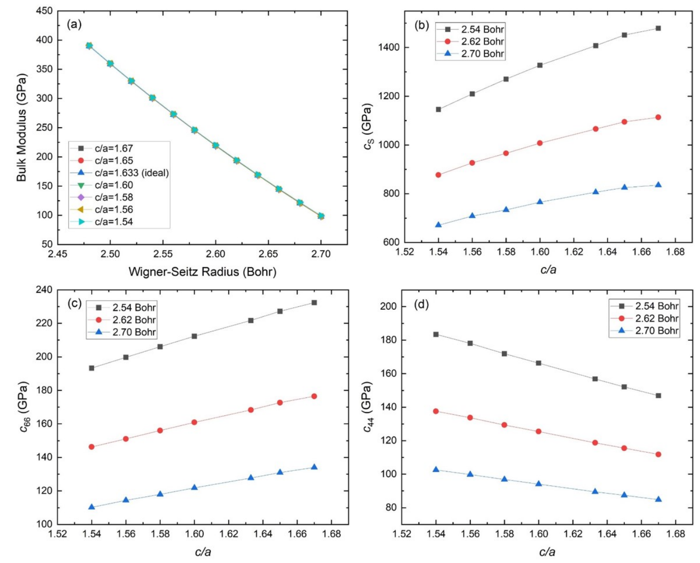

Figure S1. (**a**) Bulk modulus of hcp Fe as a function of the Wigner–Seitz radius. (**b**) c_S of hcp Fe as a function of c/a and volume. (**c**) c_{66} of hcp iron as a function of c/a and volume. (**d**) c_{44} of hcp iron as a function of c/a and volume.

Author Contributions: Conceptualization, S.-K.K. and L.V.; methodology, S.-K.K., W.L., Z.D. and L.V.; calculations Y.C. and Z.D.; writing—original draft preparation, Y.W.C.; writing—review and editing, Y.C., R.L. and L.V. All authors have read and agreed to the published version of the manuscript.

Funding: This work was supported by the Swedish Research Council (2015-5335, 2017-06474, and 2019-04971), the Swedish Foundation for Strategic Research (S14-0038 and SM16-0036), the Swedish Foundation for International Cooperation in Research and Higher Education (CH2020-8730), the Chinese Scholarship Council, the Hungarian Scientific Research Fund (OTKA 128229), the Carl Tryggers Foundation (CTS 19:212), and the National Natural Science Foundation of China (NSFC, Projects No. 51611130062 and 51374260).

Data Availability Statement: The data presented in this study are available on request from the corresponding author.

Acknowledgments: The computations were performed on resources provided by the Swedish National Infrastructure for Computing (SNIC) at the National Supercomputer Centre (NSC) in Linköping, partially funded by the Swedish Research Council through grant agreement no. 2018-05973.

Conflicts of Interest: The authors declare no conflict of interest.
